# Displacement Talbot lithography for nano-engineering of III-nitride materials

**DOI:** 10.1038/s41378-019-0101-2

**Published:** 2019-12-02

**Authors:** Pierre-Marie Coulon, Benjamin Damilano, Blandine Alloing, Pierre Chausse, Sebastian Walde, Johannes Enslin, Robert Armstrong, Stéphane Vézian, Sylvia Hagedorn, Tim Wernicke, Jean Massies, Jesus Zúñiga‐Pérez, Markus Weyers, Michael Kneissl, Philip A. Shields

**Affiliations:** 1 Department of Electrical & Electronic Engineering, University of Bath, Bath, BA2 7AY UK; 2 Université Côte d’Azur, CNRS, CRHEA, rue B. Gregory, 06560 Valbonne, France; 3 Ferdinand-Braun-Institut, Leibniz-Institut für Höchstfrequenztechnik, Gustav-Kirchhoff-Str. 4, 12489 Berlin, Germany; 4 Technische Universität Berlin, Institute of Solid State Physics, 10623 Berlin, Germany

**Keywords:** Nanowires, Nanophotonics and plasmonics, Electrical and electronic engineering

## Abstract

Nano-engineering III-nitride semiconductors offers a route to further control the optoelectronic properties, enabling novel functionalities and applications. Although a variety of lithography techniques are currently employed to nano-engineer these materials, the scalability and cost of the fabrication process can be an obstacle for large-scale manufacturing. In this paper, we report on the use of a fast, robust and flexible emerging patterning technique called Displacement Talbot lithography (DTL), to successfully nano-engineer III-nitride materials. DTL, along with its novel and unique combination with a lateral planar displacement (D^2^TL), allow the fabrication of a variety of periodic nanopatterns with a broad range of filling factors such as nanoholes, nanodots, nanorings and nanolines; all these features being achievable from one single mask. To illustrate the enormous possibilities opened by DTL/D^2^TL, dielectric and metal masks with a number of nanopatterns have been generated, allowing for the selective area growth of InGaN/GaN core-shell nanorods, the top-down plasma etching of III-nitride nanostructures, the top-down sublimation of GaN nanostructures, the hybrid top-down/bottom-up growth of AlN nanorods and GaN nanotubes, and the fabrication of nanopatterned sapphire substrates for AlN growth. Compared with their planar counterparts, these 3D nanostructures enable the reduction or filtering of structural defects and/or the enhancement of the light extraction, therefore improving the efficiency of the final device. These results, achieved on a wafer scale via DTL and upscalable to larger surfaces, have the potential to unlock the manufacturing of nano-engineered III-nitride materials.

## Nanolithography: III-nitride semiconductors

Displacement Talbot lithography enables the low-cost fabrication of a range of nanostructures from III-nitride materials. III-nitride semiconductors provide interesting and useful optical properties when formed into nanostructures, with numerous device applications. However, several key performance parameters are sometimes limited due to crystal defects, or for devices larger than the nanoscale. Now, an international group led by Philip Shields from University of Bath, UK, apply Displacement Talbot lithography, and a lateral planar displacement variation, to generate low-cost 3D nanostructures. Fabricated devices include AlN nanorods, GaN nanotubes and InGaN/GaN core-shell nanorods. This technology might enable the large-scale, low-cost fabrication of nanostructures for optoelectronic applications with enhanced light extraction, such as for light emitting diodes, and is also appropriate for other semiconductor materials.

## Introduction

III-nitride semiconductors have a crucial place in today’s optoelectronic and electronic devices^1^. In particular, III-nitride-based light-emitting diodes (LEDs) and laser diodes (LDs) have allowed efficiency breakthroughs in general illumination^2,3^, which was acknowledged by the Nobel Prize for physics in 2014 to the pioneer researchers Isamu Akasaki, Hiroshi Amano, and Shuji Nakamura “for the invention of efficient blue light-emitting diodes, which have enabled bright and energy-saving white light sources”^4,5^.

One key parameter to establish the performance of an LED is the external quantum efficiency (EQE), which represents the ratio of the number of charge carriers injected into the device to the number of photons emitted by the LED. The EQE is given by the product of the internal quantum efficiency (IQE) and light extraction efficiency (LEE). In III-nitride materials, where layers are grown on foreign substrates such as sapphire and silicon due to the limited availability and large cost of native substrates, the relatively high densities of defects generated during growth can have a dramatic impact on the IQE. In addition, the relatively large refractive index of III-nitride materials seriously limits the amount of light that can be extracted from the LED as the majority of photons will be trapped within the structure by total internal reflection. Nanostructuring these materials not only offers a route to improve the crystal quality and increase the light extraction^6,7^, but also provides an opportunity for further control of the overall device optoelectronic properties (e.g. wavelength range, lasing, doping)^8,9^, enabling novel functionalities and applications such as piezoelectric nanogenerators^10^, solar light harvesting^11^, water splitting^12^, single photon sources^13^, or intersubband devices^14^.

Compared with conventional 2D planar layers, 3D nanostructuring can, for example, reduce the dislocation density^15,16^, relieve the strain^17^, or improve the light extraction^6,7^. Nanostructuring can be implemented at various stages of the fabrication process of an LED device: at early stages prior to the growth of III-nitride layers, after the growth of an LED structure, or even at intermediate growth stages.

Patterning of substrates or III-nitride buffer layers has been widely used in early stages of the growth to reduce the formation of extended defects or block their propagation. Stripe micro-patterned sapphire and silicon substrates, with or without a dielectric mask, have been successfully exploited to achieve high-quality polar, semi-polar and non-polar layers with a process that is often referred to as ‘selective area growth’ (SAG) or ‘epitaxial lateral overgrowth’ (ELO) or a combination of both^18,19^. The geometrical shape of the pattern can also enhance light extraction by scattering or redirecting the light at the roughened substrate/III-nitride interface^20^. Finally, sub-micron-scale nanopatterned sapphire substrates (nano-PSS) can enhance crystal quality and light extraction^21^, whilst simultaneously reducing the buffer layer thickness with the benefit of further lowering production costs^22^.

Further improvement to enhance light extraction can be accomplished once the LED structure has been grown, either by texturing the surface of the LED^23,24^, shaping the LED chip^25^, or encapsulating the LED chip^26^. Surface texturing helps to break up the guided modes confined within the LED structure by creating a surface that randomizes the propagation of light and increases the probablility of photons escaping. Although surface roughening, either on the p-GaN top surface^23^, or at the n-side-up GaN surface^24^, has been successfully applied to increase light extraction, it provides poor control over the direction of the emitted light, resulting in Lambertian radiation patterns. Instead, the use of a periodic pattern provides a route to control the directionality^27^. In particular, photonic crystals, at the surface or embedded within the LEDs can increase the extraction efficiency, improve the directionality and enhance the IQE due to the Purcell effect^28,29^. In these architectures, the LED performance and directionality depends on the type, depth, filling factor and pitch of the photonic crystal along with the thickness of the epitaxial layers^30^.

III-nitride nanorod LEDs are an alternative to reducing the dislocation density and improving light extraction in 2D layers^7–9^. III-nitrides nanostructures can be fabricated via either a top-down or bottom-up approach. While the top-down approach typically involves nanopatterning and subsequent etching^16,31^, the bottom-up growth of nanostructures does not necessarily require a post-patterning process^32^. However, to control their position, height and size uniformity, and also to reach sufficiently homogenous optical properties, significant effort has focused on the SAG on patterned substrates or templates^33,34^.

Therefore, there are a number of different perspectives that provide a strong motivation for a nanolithography technique that is capable of nano-engineering III-nitride materials. However, a key requirement of the technique for its widespread use would be the capability to create large-scale nanopatterns at low cost, enabling a reduction of its impact on the overall fabrication cost. We have therefore explored Displacement Talbot lithography (DTL) as a fast and robust emerging patterning technique that can pattern rough and bowed wafers with features down to 100 nm on large areas, e.g. 4-inch wafers^35^. As a nanolithography process, it is considerably lower cost than electron beam lithography (EBL), and competes with nanoimprint lithography (NIL) and laser interference lithography (IL) as a wafer-scale process. However, it has advantages over both these latter processes, such as a low sensitivity to substrate surface defects, no issues with master lifetime, and a high system stability, which is of particular interest for the manufacture of nano-engineered semiconductors, including III-nitrides materials. To date, several reports using DTL have been published over the last few years, mainly focusing on resist patterning^35–38^, and the fabrication of metal nanoparticles^39^, high-aspect ratio Si nanostructures^40^ or high resolution gratings^41^.

This paper is the first comprehensive report on the use of DTL to successfully nano-engineer a wide range of III-nitride and related materials. After introducing the overall fabrication process, the DTL and extended D^2^TL nanopatterning capabilities are presented with the fabrication of nanoholes, nanodots, nanorings and nanolines in positive and/or negative resists. The resist patterns are transferred into dielectric or metal masks by etching or lift-off for use as a mask for bottom-up growth, top-down etching or a combination of both. Examples of such nano-engineering applied to III-nitride materials will be given, such as the bottom-up selective area growth of InGaN/GaN nanorods, the top-down etching of a variety of nanostructures from III-nitride template or LED structures, the top-down sublimation of high-aspect ratio GaN nanoholes and nanorods, and the hybrid top-down/bottom-up of AlN nanorod and GaN nanotubes. The fabrication of nanopatterned sapphire substrates and successful overgrowth and coalescence of an AlN layer will also be presented. Compared with the planar approach, these 3D nanostructures enable the reduction of defects and/or the enhancement of light extraction, therefore improving the efficiency of the final device.

## Results and discussion

**Fig. 1:**
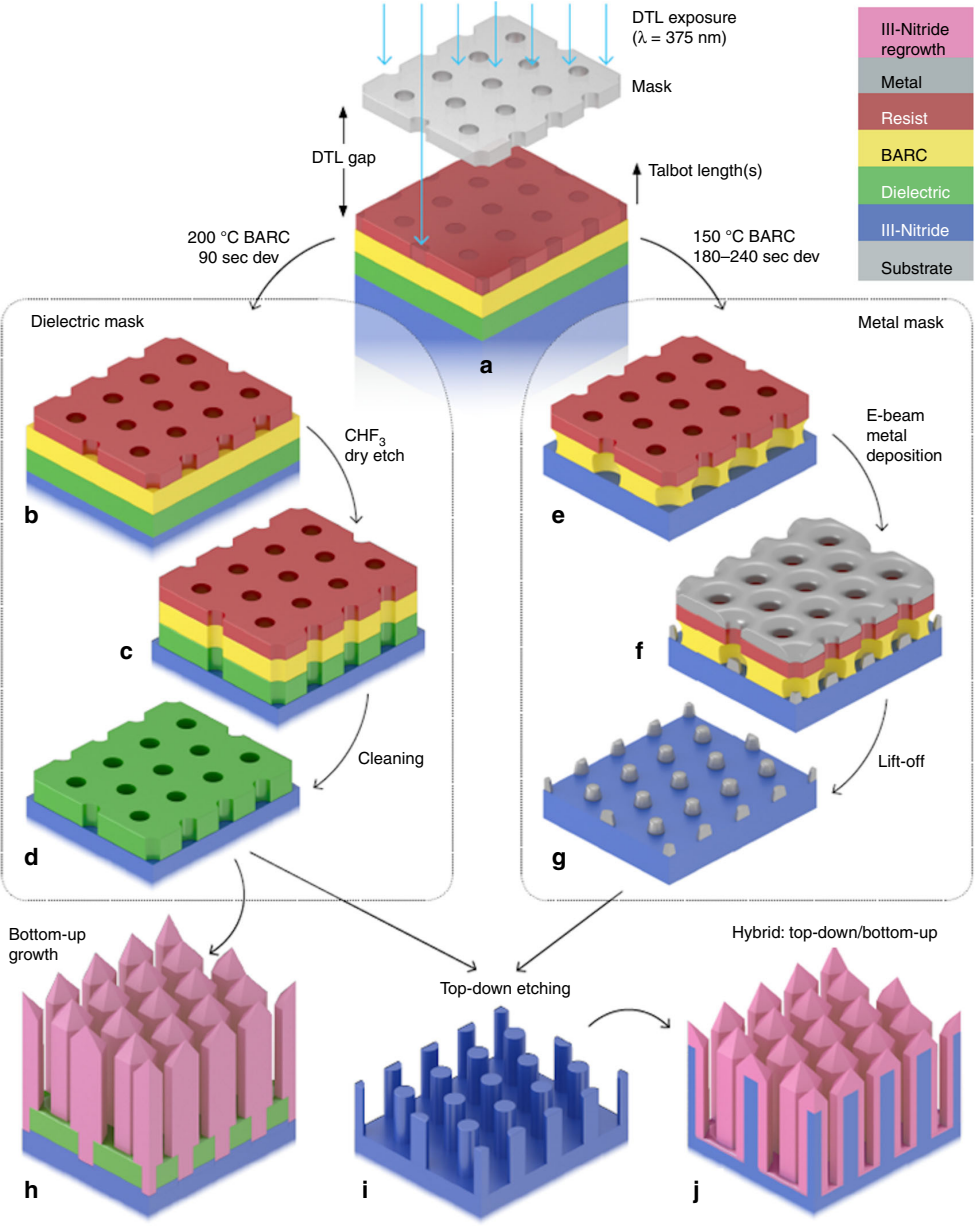
Schematic of the III-nitride nano-engineering process via DTL. **a** Coating of III-nitride wafer or substrate and DTL exposure of the resist. **b**, **c**, **d** For a bottom anti-reflective coating (BARC) layer hard-baked at 200 °C, the DTL pattern is first developed, then transferred via dry etching into the BARC and dielectric layer and cleaned. **e**, **f**, **g** For a BARC layer soft-baked at 150 °C, the DTL pattern is developed and used to create an undercut profile prior to metal evaporation and lift-off. **h** Dielectric mask for bottom-up growth. **i** Dielectric and metal mask for top-down etching. Pillars illustrated but other pattern/geometries achievable. **j** Combination of top-down etching and bottom-up growth

III-nitrides are robust materials that can be difficult to plasma etch. For example, III-nitrides etch at much slower rates than conventional III–V compound semiconductors^42^. They generally require a chlorine-based plasma and in some cases high temperatures^42,43^. Therefore, photoresists are unsuitable, especially when thinner resists are required to improve the resolution, a necessary requirement for nano-engineering. Instead, the use of a hard mask, either dielectric or metallic is preferred; in particular, a metal mask will be essential when deep etching or high-aspect-ratio nanostructures are desired^43,44^. Moreover, the selective area growth of III-nitride nanostructures requires inert and robust material such as SiN_x_, SiO_x_ or TiN_x_^15,33,34^. The nanostructured dielectric layer enables enhanced adatom diffusion and preferential deposition of species on the III-nitride window. Therefore, one would ideally require a lithography method to fabricate equally well both dielectric and metal masks, which are required to nano-engineer III-nitrides. Figure 1 shows schematically the processing steps for these two mask types. More specific details of each fabrication step can be found in the ‘Materials and methods’ section.

### Displacement Talbot lithography

**Fig. 2:**
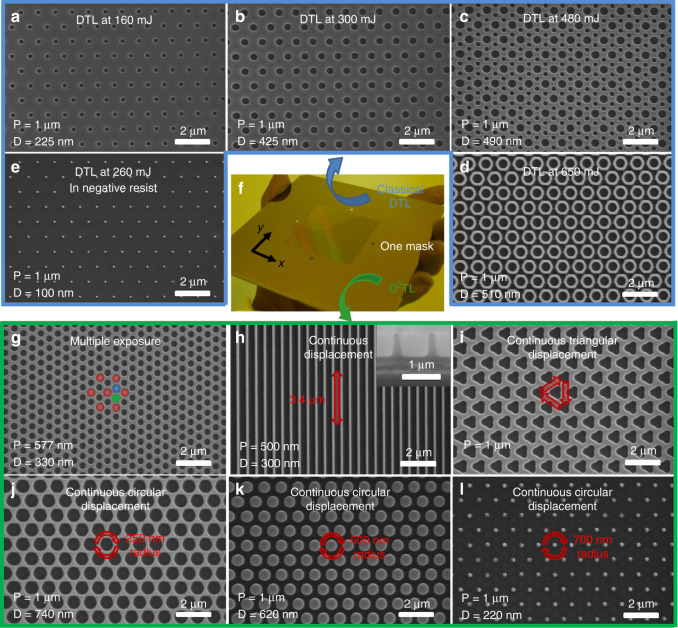
DTL (blue box) and D^2^TL (green box) nanopatterns created from a 1 µm pitch 550 nm opening mask. Developed positive photoresist after classical DTL at **a** 160 mJ/cm^2^, **b** 300 mJ/cm^2^, **c** 480 mJ/cm^2^ and **d** 650 mJ/cm^2^. Developed negative photoresist after classical DTL at **e** 260 mJ/cm^2^. **f** 1 µm pitch 550 nm opening amplitude mask used to produce all the patterns presented in Fig. 2. Developed positive photoresist after D^2^TL for **g** three exposures, at *x* = 0, *y* = 0, *x* = 500 nm, *y* = 289 nm and *x* = 500 nm, *y* = 289 nm. **h** 3.4 µm continuous linear displacement. **i** triangular displacement **j** 250 nm radius circular displacement. **k** 500 nm radius circular displacement. **l** 750 nm radius circular displacement

The Talbot effect, or self-imaging, is the effect of creating a periodic three-dimensional interference pattern when a periodic mask is illuminated by a coherent light^45^. The interference pattern reproduces itself when *z* is a multiple of the ‘Talbot length’. By displacing the wafer along the *z*-axis of illumination over integer spatial periods, the low depth of field of conventional Talbot lithography is overcome^35^. Figure 2a–d shows experimental patterns achieved for various doses in a positive resist for a 1-µm-pitch mask with a hexagonal arrangement of 550 nm diameter holes, which is shown in Fig. 2f. The nanohole openings increase in diameter from ~225 to ~425 nm with an increase in the exposure dose from 160 to 300 mJ/cm^2^. For doses >300 mJ/cm^2^, a secondary pattern of holes appears (Fig. 2c) and merges to create a nanoring pattern having an inner diameter of ~520 nm and a wall width of ~150 nm (Fig. 2d). Alternatively, using a negative resist creates arrays of nanodots (Fig. 2e). Our previous paper explores, by means of simulations and experiments, the resolution limit of DTL as a function of the resist employed, the configuration of the mask and the wavelength of illumination^38^.

The novel introduction of lateral displacements either during a single DTL exposure or between multiple exposures greatly extends the range of patterns achievable by a single mask. We call this technique ‘Double Displacement Talbot lithography’ (D^2^TL). Figure 2g–l shows examples of additional patterns in positive resist from the same mask. A reduction of the pitch from 1 µm to ~577 nm is obtained with multiple exposures. In Fig. 2g, the red dots represent the first exposure and the blue and green dots correspond to a second and third exposure. Continuous displacements during a single exposure can form nanogratings (via displacement along the *x*- or *y*-axis (Fig. 2h)), triangular features (via a triangular displacement (Fig. 2i)), or a wider range of circular features that cannot be obtained through simple DTL (via circular displacements of different radii (Fig. 2j–l)). The D^2^TL patterns presented in Fig. 2g–i represent a small sample of the D^2^TL capabilities, since the technique can also generate periodic arrays of complex features. Further technical details of D^2^TL are to be published separately^46^.

Therefore, for one mask configuration, a broad variety of features and configurations has been created via DTL and D^2^TL. Achieving the various patterns presented in Fig. 2 by NIL would require up to 11 masters. This highlights the tremendous flexibility of the technique to pattern large areas with a much lower fabrication cost than normally associated with other nanopatterning techniques.

### Dielectric and metal mask for selective area growth and etching

**Fig. 3:**
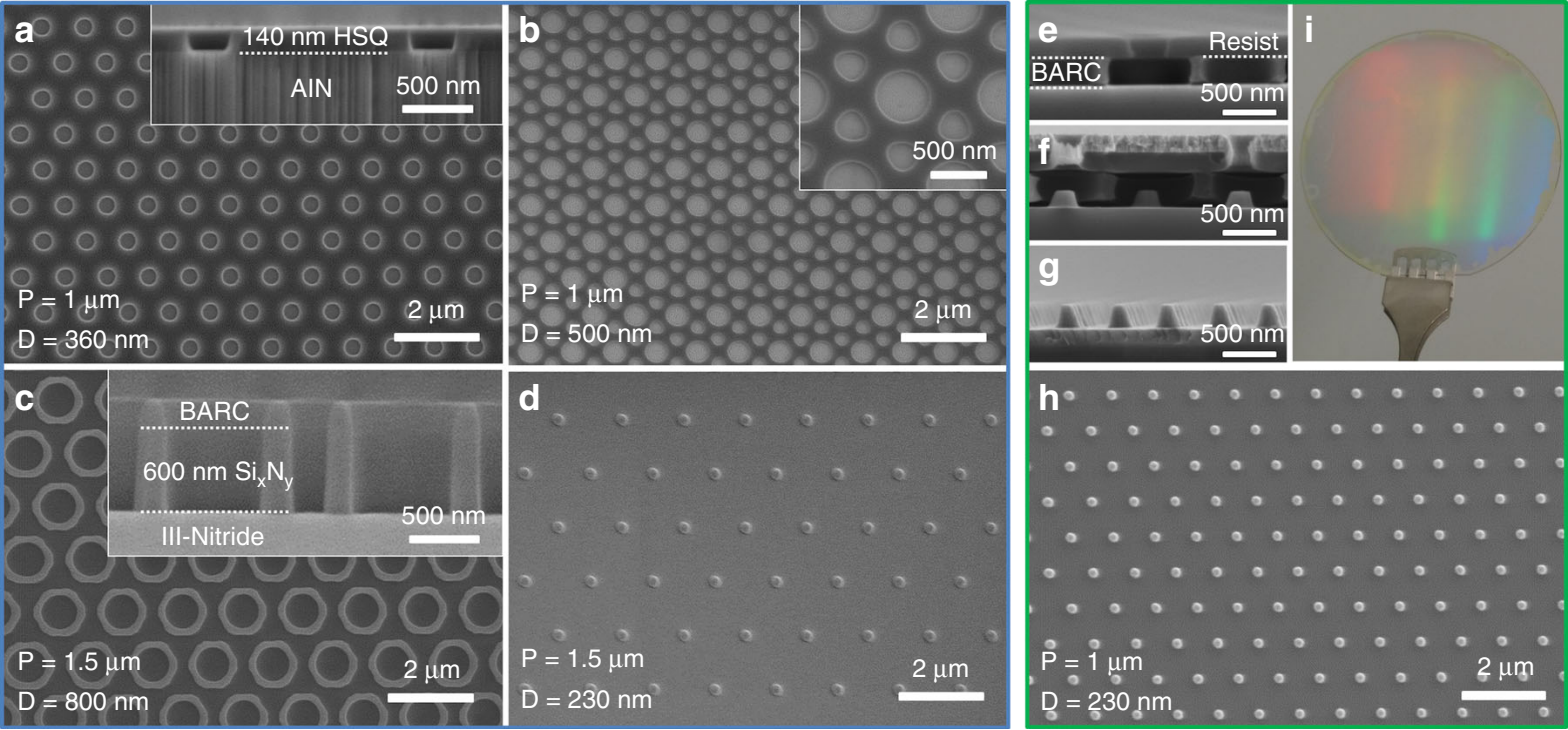
Dielectric mask (blue box) and metal mask (green box). **a** 1 µm pitch nanoholes opening in 140 nm hydrogen silsesquioxane (HSQ). **b** Double periodicity of nanoholes opening in 30 nm HSQ, with a primary pattern of 1 µm pitch. **c** 1.5 µm pitch rings in 600 nm SiN_x_. **d** 1.5 µm pitch dots in 30 nm HSQ. **e**–**g** SEM cross-section image after exposure and development of the positive resist, after metal deposition and after lift-off. **h** SEM plan-view image of a hexagonal array of metal dots after lift-off. **i** Photograph of 2-inch GaN template with metal dot array on its surface

Dielectric and metal masks can be produced from any of the previous nanopatterns presented in Fig. 2 or other configurations of the mask. Figure 3a–d presents various hexagonal arrays of periodic nanostructures transferred in a dielectric layer via inductively coupled plasma (ICP) etching. Arrays of nanoholes (Fig. 3a, b), nanorings (Fig. 3c) and nanodots (Fig. 3d) are successfully created on any dielectric layer, up to a thickness of 600–700 nm (Fig. 3d). Alternatively, metal masks are also fabricated via lift-off, as shown in Figs. 1e–g and 3e–i. Compared with dielectric material, metals generally possess a better selectivity to III-nitrides, which will facilitate deeper etching into the layer.

As DTL is a non-contact patterning technique, the fabrication of a dielectric mask or a metal mask is independent of the surface roughness of the III-nitride layer and the wafer bow. In contrast, contact patterning such as NIL can induce serious damage (e.g. cracks or fractures) on bowed and fragile wafers such as GaN on silicon. Hence, DTL provides a robust and reproducible fabrication process for selective area growth and top-down etching of III-nitrides at a wafer scale. Note that the fabrication process employed to create a dielectric or a metal mask can be easily transferred on any substrate (e.g. Si, Al_2_O_3_…) or other semiconductor materials (e.g. GaAs, InP, ZnO…).

### Bottom-up selective area growth

**Fig. 4:**
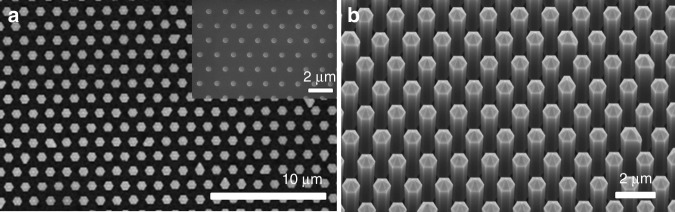
InGaN/GaN core-shell nanorods. **a** SEM plan-view image and **b** tilted image of 4.2 µm height 800 nm diameter core-shell structure with three InGaN QWs. The inset in Fig. 4a displays the initial dielectric mask composed of 350 nm hole openings in 30 nm HSQ with a 1.5 µm pitch

Nanohole openings in a relatively thin dielectric layer are the most common configuration to perform selective area growth of nanostructures such as nanopyramids or nanorods^7,8,15,33^. Fig. 4 displays InGaN/GaN core-shell structures grown by MOVPE on patterned Ga-polar GaN on sapphire template. The initial nanopatterning performed via DTL allowed the successful control of the position, diameter and density of the nanorods while optimized MOVPE growth conditions enabled the vertical growth of nanostructures and additional control on the diameter^15,47^.

The selective area growth of GaN nanorods and InGaN/GaN-based core-shell structures have been widely investigated over the past decades, with some promising results and properties, such as a low turn on voltage, low series resistance, short carrier lifetime^48^, the potential to achieve monolithic RGB emission^49^ and a control over the far-field emission pattern^50^, which is particularly attractive for specific applications such as solid state lighting, visible light communication or µ-LEDs. However, to commercialize these structures, the growth and device fabrication must be performed on a large wafer scale. Many reports employ EBL to pattern a dielectric mask^7,8,15,47,49^, which, despite the high resolution, often limits the area over which nanostructures can be grown. Alternatively, NIL and IL provide wafer-scale nanopatterning with reasonably high resolution^7,8,33^. However, NIL is sensitive to surface defects and the lifetime of the master is limited, whilst IL requires a high system stability. Here we demonstrate that DTL can be successfully used for such a purpose which could help to further reduce the cost of InGaN/GaN core-shell LEDs enabling manufacturing^51,52^.

### Top-down fabrication of nanostructures

#### III-nitride dry etching

**Fig. 5:**
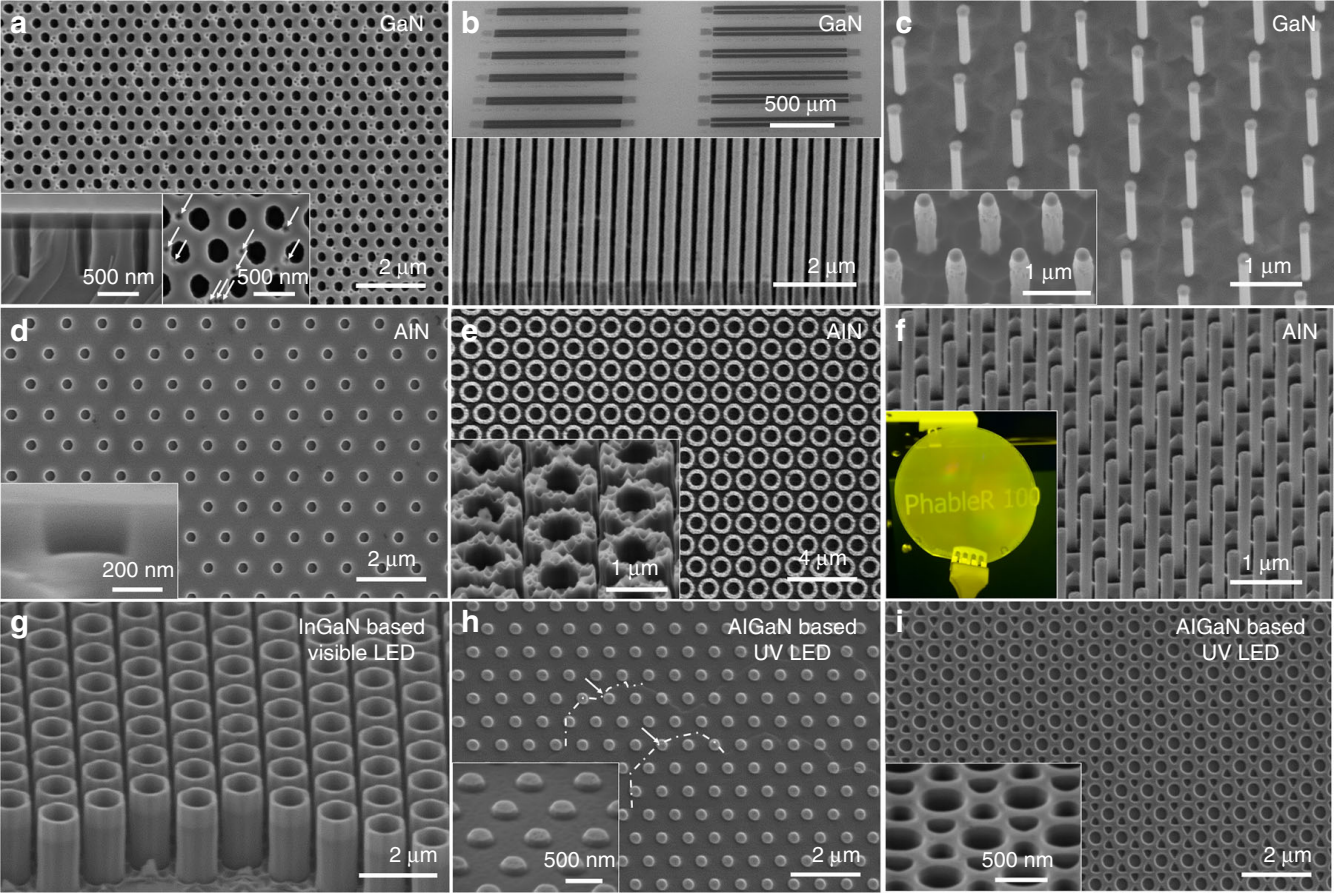
III-nitride top-down etched nanostructures. **a** Plan-view SEM image of ~600 nm depth GaN nanoholes. **b** 45° tilt SEM image of ~800 nm depth GaN nanolines (bottom) and related GaN grating couplers (top). **c** 10° tilted SEM image of ~3 µm height GaN nanorods after ICP dry etch (inset) and KOH-based wet etch at 60 °C. **d** Plan-view SEM image of ~150 nm depth AlN nanoholes. Inset shows a cross-section along one single nanohole. **e** Plan-view SEM image of ~800 nm height AlN nanotubes. **f** 45° tilt SEM image of ~1.8 µm height AlN nanorods after ICP dry etch and AZ400K wet etch at RT and associated photograph of 2-inch AlN nanorod template in inset. **g** 45° tilt SEM image of ~2.4 µm height axial InGaN/GaN nanotubes. **h** Plan-view SEM image of ~100 nm depth p-GaN/AlGaN nanopillars at the surface of an AlGaN based UV LED. **i** Plan-view SEM image of double periodicity of nanoholes at the surface of an AlGaN based UV LED with a ~250 nm depth

From the various DTL nanopatterns presented in Fig. 2, a broad range of III-nitride nanostructures have been obtained via chlorine-based ICP dry etching. Depending on the application targeted, the thickness, nature and configuration of the mask were optimized to achieve a wide range of periods and aspect ratios. Figure 5a–c presents arrays of GaN nanoholes, nanolines and nanorods. Figure 5d–f similarly shows arrays of AlN nanoholes, nanotubes and nanorods. Finally, Fig. 5g–i shows various nanopatterns transferred in visible and UV based III-nitride LED structures to form axial InGaN/GaN nanotubes, p-GaN/AlGaN nanopillars and AlGaN nanoholes with two periodic sizes. Nanostructures with a relatively high-aspect ratio can be achieved, such as those in Fig. 5c, f and g with values of 21.4, 7.8 and 2.2, respectively. Note that a difference in etch rate is commonly observed between III-nitride materials and explained by the higher binding energy of AlN compared with GaN and InN^53^. This will lead to a decrease of the etch rate with an increase in Al content^54^.

A common feature of the nanostructures presented in Fig. 5a–g is the presence of a relatively straight sidewall profile, regardless of the nature of the mask or of the III-nitride material. The sidewall profile of the nanostructures can be tuned by changing the plasma properties such as the pressure, the temperature, the RF power and ICP power. More details on the etching recipes and the impact of the plasma properties can be found in the ‘Materials and methods’ section and previous publications^43,44,55^. It is also possible to further tune the dimensions and profile of the nanostructures by further wet etching the nanostructure in a KOH-based solution. Figure 5c shows GaN nanorods before and after wet etching. As already reported in the literature, KOH-based wet etching helps to remove plasma etch damage, improves the sidewall profile and controls the geometry^16,56^.

These results also demonstrate the robustness of the DTL based fabrication process to high surface roughness such as pits (inset of Fig. 5a, e) or terraces (partially highlighted by a dash line in Fig. 5h). The AlN nanotubes in Fig. 5e were fabricated despite the high roughness of the initial template, as indicated on the top of the tubes after dry etching and the removal of the etch mask.

There are multiple purposes for creating nanostructures having various dimensions, geometry and filling factor. Firstly, light extraction can be improved by nano-texturing the surface of LEDs with shallow nanostructures. The nano-textured AlGaN based UVB LED surfaces presented in Fig. 5h, i will not only scatter the light and improve light extraction but also reduce the absorption within the thin top p-GaN layer that is commonly needed in UVB LEDs to get a low resistance contact. Secondly, light extraction can also be improved and directionality controlled by creating photonic crystals from dense arrays of nanoholes and nanopillars, even to the extent of achieving lasing^28,29,57^. Indeed, the use of multiple exposures via D^2^TL (Fig. 2g) is a promising approach to attain the small pitches required for photonic crystals at short wavelengths on a wafer scale. Thirdly, III-nitride photonic circuits can be created by combining grating couplers comprising dense arrays of nanolines (top SEM image in Fig. 5b) with other nano/microstructures^58,59^. Fourthly, the emission across the visible or the UV spectrum can be tuned by engineering the strain in an embedded active region through the control of the nanorod diameter^60^. Finally, nanolaser cavities, where the light is confined either between the top and bottom facet or within the circumference of the structure, can be constructed from high-aspect-ratio nanostructures such as the rods and tubes (Fig. 5c, f, g)^55,56^.

#### GaN selective area sublimation

**Fig. 6:**
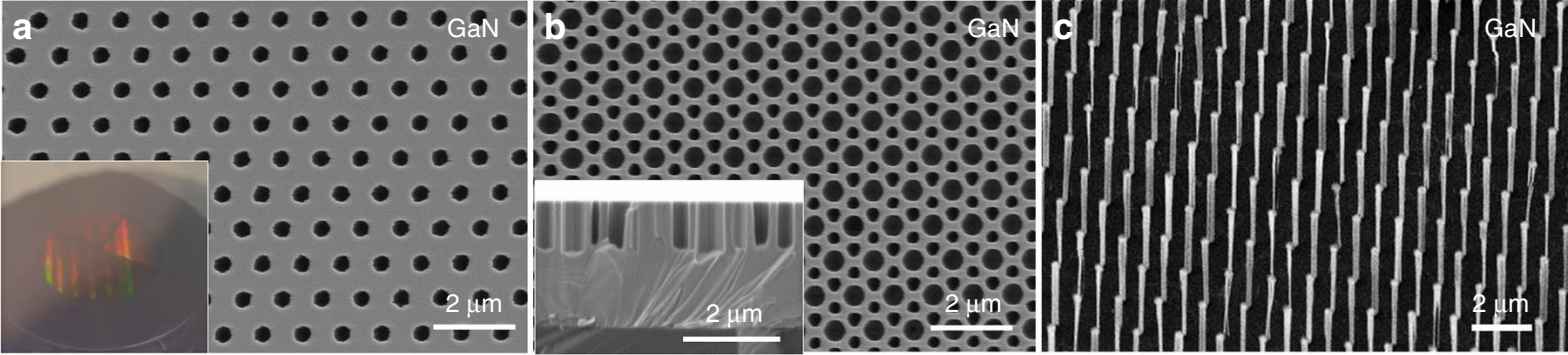
GaN selective area sublimation. **a** SEM plan-view image of 1 µm height holes in GaN. **b** SEM plan-view and cross-section (inset) images of double periodicity holes in GaN of ~1 µm height. **c** SEM-tilted image of 4-µm height 100–400-nm-diameter GaN nanorods. Inset in (**a**) shows a 2-inch GaN wafer during the sublimation process with one configuration of nanohole per quarter

Sublimation has been recently proposed as a simple top-down route to form nanostructures such as nanorods, nanopyramids, InGaN quantum discs or nanoporous material from GaN-based material without introducing the damage that occurs in dry etching^61–63^. By protecting the GaN surface with a thermally resistant dielectric layer and then annealing the sample under vacuum and sufficiently high temperatures, selective area sublimation of GaN can be carried out through the apertures of the mask. Figure 6 displays top-down selective area sublimation experiments performed on a Ga-polar GaN-on-sapphire template with various nanopatterns etched into a 50 nm thick SiN_x_ mask. After 3 h of sublimation within a UHV chamber and SiN_x_ removal in buffered oxide etch (BOE), 1 µm height nanoholes are obtained (Fig. 6a, b) while 4 µm height nanorods are attained after 10 h. As expected, the initial DTL pattern provides highly organised nanostructures at a wafer scale (see inset in Fig. 6a). Both the nanoholes and the nanorods show relatively straight sidewall profiles and a horizontal to vertical sublimation rate of 2–3% due to the high thermal stability of non-polar GaN planes in vacuum.

By comparing top-down approaches, selective area sublimation provides vertical sidewalls, no etching damage, and a perfect mask selectivity, enabling the formation of high-aspect ratio nanoholes or nanorods with a very thin dielectric mask. However, sublimation is sensitive to structural defects^64^, occurs not only vertically but also laterally, and is only suitable for GaN and InGaN materials; not AlGaN with more than 10% Al^65^. Therefore, although promising for photonic applications and the nanostructuring of GaN materials^62,63,65,66^, ICP dry etching provides more flexibility on the type of materials that can be patterned and on the nanostructure profile.

#### Combined top-down/bottom-up processing

**Fig. 7:**
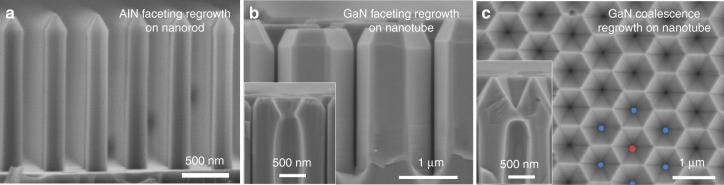
III-nitride hybrid top-down/bottom-up nanostructures. **a** SEM cross-section image of AlN nanorods after AlN MOVPE regrowth. **b** SEM cross-section image of GaN nanotubes after GaN MOVPE regrowth. **c** SEM plan-view image of an array of GaN nanopyramidal pits after GaN MOVPE regrowth. Both inset SEM images in (**b**) and (**c**) show a cleaved cross-section in the middle of the tube

The hybrid top-down/bottom-up approach combines the fabrication of highly-uniform and organized nanostructures, such as those presented in Figs. 5 and 6, with an additional regrowth step, similar to the selective area growth in Fig. 4. Depending on the growth conditions, the initial pattern configuration and the dimensions of the nanostructures, the regrowth of III-nitride material can lead either to the formation of a planar 2D layer or to the formation of 3D nanostructures with well-defined crystallographic facets. The latter is presented in Fig. 7 where images are shown of AlN regrowth performed on AlN etched nanorods (Fig. 7a), and of GaN regrowth on GaN etched nanotubes for two growth conditions (Fig. 7b and c). For some growth conditions^16,44,67,68^, coalescence is inhibited, and straight and smooth non-polar sidewall facets are formed for both GaN and AlN (Fig. 7a, b). For different growth conditions, coalescence is induced, for example to create dense arrays of nanopyramidal pits from the top of GaN nanotubes (Fig. 7c).

As the growth of AlN nanorods by selective area growth has yet to be achieved due to the high sticking coefficient and the low diffusion length of Al atoms^69^, the combination of top-down etching and bottom-up MOVPE growth represents a reliable approach to fabricate uniform and homogenous arrays of AlN nanorods, as shown in Fig. 7a. These are of particular interest for the subsequent growth of deep-UV core-shell structures^70^. The ability to fabricate further geometries, such as nanotubes (Fig. 7b) or nanopyramidal pits (Fig. 7c) opens the possibility to grow active regions such as quantum wells on specific facets or quantum dots in preferential locations. Therefore, compared with conventional MOVPE selective area growth alone, the combination of top-down etching and bottom-up regrowth enables the exploration of a broader range of nano-light-emitting architectures.

**Fig. 8:**
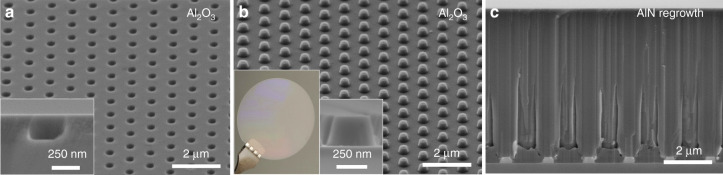
AlN regrowth on nanopatterned sapphire substrate. **a** 45° tilt SEM image of nanoholes in sapphire substrate with an inset in a high-magnification cross-section image of the Al_2_O_3_ nanohole. **b** 45° tilt SEM image of nanopillars in sapphire substrate with an inset in a photograph of a 2-inch nPSS and a high magnification cross-section image of the Al_2_O_3_ nanopillars. **c** SEM cross-section image of 6-µm-thick AlN layer grown on nanopillar patterned sapphire substrate

III-nitride growth can also be performed on nanostructured foreign substrates following the same fabrication process as for III-nitrides. Nanopatterned sapphire substrates (nPSS) have been obtained on 2-inch wafers either with an array of nanoholes (Fig. 8a) or nanopillars (Fig. 8b). These features are uniform across the 2-inch wafer (Fig. 8b), with a flat top *c*-plane preserved after fabrication (inset in Fig. 8a, b). Figure 8c shows the growth and successful coalescence of AlN layers carried out on the pillar-nPSS wafer, following growth conditions previously reported^22^.

Compared with wet etching of sapphire, which is facet dependent and thus limited in depth for small features, the combination of a thick dielectric and/or metal mask with chlorine-based top-down dry etching enable to tune both, the etch depth and sidewall profile. While the NIL process becomes less trivial for thick resist, the use of DTL allows the patterning of thick resist at the nanoscale and thus, to create thick SiN_x_ mask.

By improving the crystal quality of the layers and the light extraction of the final device^21,22^, the nanostructuring of a sapphire substrate is of particular interest to improve the efficiency of DUV LEDs^71^. The use of DTL/D^2^TL constitutes a reliable and cost effective option to create nPSS with a broad range of configurations.

## Conclusions

This work demonstrates the high potential of DTL for the large-scale nano-engineering of III-nitride materials for numerous optoelectronic applications as a fast, robust and scalable process. In particular, the novel and unique implementation of lateral planar displacement (D^2^TL) considerably extends the flexibility of the technique. As such, a broad variety of features and configurations have been obtained in positive and negative resist. DTL has been successfully used to create dielectric and metal masks, and to nano-engineer various III-nitride layers via selective area growth, selective area sublimation, top-down etching and hybrid top-down/bottom-up growth. The use of nanostructures or nano-textured surfaces is the key to achieve higher efficiency III-nitride LEDs mainly thanks to defect reduction and light extraction enhancement. It is also important to point out that the fabrication process can be transferred to any semiconductor materials such as other smaller band gap III–V materials (GaAs, InGaAs, InP…).

Despite the broad range of configurations and feature sizes already demonstrated in this paper, the capabilities of DTL in terms of resolution can be extended with a shorter wavelength illumination source. Recently, Eulitha demonstrated wafer-scale sub-wavelength features with a 266 nm source^37^. Therefore, by combining the D^2^TL approach with a shorter illumination wavelength, low pitches < 250 nm could be achieved on a wafer scale, which would be of major interest to create photonic crystals in III-nitride-based UV LEDs and further improve their efficiency^71,72^.

## Materials and methods

### DTL patterning

All DTL patternings have been performed on 2-inch wafers (Fig. 1a). A stack of two layers was spin-coated at 3000 rpm to obtain a ~270 nm bottom anti-reflective coating (BARC) (Wide 30 W—Brewer Science) layer thickness, followed by either a layer of high-contrast positive resist (Dow® Ultra-i 123 diluted with Dow® EC11 solvent) or a layer of negative resist (AZ® 15 NXT diluted with AZ® Edge Bead Remover (EBR) solvent with a 7:12 ratio by weight). The baking temperature is a critical parameter for the BARC processing as it determines the rate at which the BARC develops. A bake at 150 °C enables a wet-developable process and thus to create an undercut profile (Fig. 1e and 3e). A bake at 200 °C fully cures the BARC, making it insoluble in a developer (Fig. 1b). DTL (PhableR 100, Eulitha) was then used to expose the resist with a coherent 375 nm light source with an energy density of 1 mW.cm^−2^ (Fig. 1a). Various masks have been employed: two hexagonal amplitude masks, one with a 1.5 μm pitch with 800 nm diameter circular openings, and another with a 1 μm pitch with 550 nm openings, and two phase mask, one with a 500 nm pitch with 300 nm diameter circular openings, and another with lines spaced by 800 nm with a 62% filling factor. The Talbot length associated with those masks is 8.81 μm, 3.80 μm, 750 nm and 3.21 μm, respectively. Details of the calculation can be found in other publications^35^. The initial gap between the mask and the wafer was set to 150 μm. A Gaussian velocity integration was applied and eight Talbot lengths travel distance has been chosen to assure a homogenous integration on several Talbot motifs. After a certain exposure time (which defines the exposure dose), the sample was baked for 1 min 30 s at 120 °C on a hot plate. The wafer with a positive resist was developed in MF-CD-26 for 90–240 s (depending on the mask fabrication), the one with a negative resist in AZ 726 for 30 s. Finally, the wafer was rinsed with deionized water and dried with nitrogen.

### Dielectric mask fabrication

Materials such as hydrogen silsesquioxane (HSQ) and silicon nitride (SiN_x_) were used as a dielectric mask. Prior to DTL patterning, HSQ was spin-coated on 2-inch wafers at 3000 rpm and baked from 150 to 450 °C in 100 °C increments or SiN_x_ was deposited by plasma enhanced chemical vapour deposition (PECVD). The patterns created in the resist by DTL/D^2^TL (Figs. 1b and 2a–l) were transferred into the dielectric material (Figs. 1c and 3a–d) via an inductively coupled plasma (ICP) dry etch system (Oxford Instruments System 100 Cobra). The experiments were performed with a CHF_3_ chemistry of 25 sccm, a temperature set to 20 °C, a pressure of 8 mTorr, 50 W RF power and 300 W ICP source power, resulting in a etch rate of ~50 nm/min. The etching time was adjusted as a function of the thickness of the dielectric mask. The resulting transferred pattern was then cleaned in a piranha solution (H_2_SO_4_:H_2_O_2_ 3:1) and oxygen plasma (Fig. 1d).

### Metal mask fabrication

The undercut profile created in the BARC (cured at 150 °C) after exposure and development (Figs. 1e and 2e) was employed as a lift-off layer. Two-hundred nanometers of Ni was deposited via e-beam evaporation to produce metal masks in the circular openings at the surface of the wafer (Figs. 1f and 2f). Subsequent lift-off was achieved by soaking the wafer in MF-CD-26 developer. Finally, wafers were cleaned in a 2 min reactive-ion etching (RIE) oxygen plasma to remove any BARC residue (Figs. 1g and 2g–i).

### Bottom-up growth of GaN nanorods

The selective area growth of InGaN/GaN core-shell nanorods has been carried out in a showerhead MOCVD reactor. The GaN core has been grown under continuous flow mode with the following conditions: A carrier gas mixture of N_2_ and H_2_ with H_2_/N_2_ = 2, a temperature of 1200 °C, a total reactor pressure of 100 mbar, and the TMGa and NH_3_ flow rates fixed at 80 sccm. Other details about the growth conditions and optimization can be found in previous publications^15,47^. On the GaN nanorods, five periods of InGaN/GaN QWs have been deposited using standard QW growth conditions, TMIn, TEGa and NH_3_ have been used as precursors, pressure has been fixed at 400 mbar and the growth temperature set between 850 and 980 °C for QWs and GaN barriers, respectively.

### Top-down etching of III-nitride materials

An ICP dry etch system was used to create nanostructures in various materials including GaN, AlN, III-nitride LEDs structures and sapphire substrates. In the case of III-nitrides, the experiments were performed with a Cl_2_/Ar chemistry of 50/10 sccm, a temperature of 150 °C, a pressure set between 9 and 15 mTorr, a RF power set between 80 and 120 W, and 800 W ICP source power. More details can be found in previous publications^16,43,44,55^. For sapphire substrates, the experiments were performed with a Cl_2_/BCl_3_/Ar chemistry of 5/50/5 sccm, a temperature of 5 °C, a pressure of 8 mTorr, 100 W RF power and 600 W ICP source power. Finally, the masks were etched away in aqua-regia solution (HCl:HNO_3_, 3:1) for metal masks, and in BOE 5:1 for dielectric masks.

### Sublimation of III-nitride materials

The samples used for the selective area sublimation were grown on 2-inch c-plane (0001) sapphire substrates by MOCVD in a 7 × 2-inch close-coupled showerhead Aixtron reactor. A 2 µm non-intentionally doped GaN layer was first grown followed by a 2 µm Si-doped (5 × 10^18^ cm^−3^) GaN layer. The first 2 µm of GaN are undoped in order to favour the coalescence of the layer after an initial 3D growth mode to reduce the threading dislocation density. DTL was used to pattern a SiN_x_ or a SiO_x_ dielectric mask with, respectively, holes (Fig. 3b) or dots (Fig. 3d). The samples were annealed under vacuum in a MBE chamber during 3 h at 910 °C and 10 h at 940 °C for the samples with the hole pattern and the dot pattern, respectively. More details can be found in previous publications^61–63^.

### Hybrid top-down/bottom-up

The III-nitride bottom-up regrowth was carried out in a 1 × 2″ horizontal Aixtron MOVPE reactor. The growth conditions for AlN faceting on nanorod were the following: a temperature of 1100 °C, a pressure of 20 mbar, 10 sccm in TMAl flow rate, 4000 sccm in NH_3_ flow rate, and H_2_ as the carrier gas. GaN regrowth was performed at a temperature of 920 °C (Fig. 7b) or 820 °C (Fig. 7c), a pressure of 100 mbar, 8 sccm in TMGa flow rate, 2800 sccm in NH_3_ flow rate, and H_2_ as the carrier gas. More details can be found in previous publications^16,44,67,68^.

AlN overgrowth on nanopillar-nPSS was done in an AIX2400G3HT MOVPE planetary reactor with a capability of 11 × 2-inch wafers with standard TMAl and NH_3_ precursors. Pressure was fixed at 50 mbar and H_2_ served as carrier gas. A 50-nm-thick nucleation layer was deposited at 980 °C with a V/III ratio of 4000. After nucleation, the temperature was increased to 1380 °C with a V/III ratio of 30, followed by a decrease to 1180 °C with the same V/III ratio. More details on the growth process can be found in a previous publication^22^.

### SEM imaging

Scanning electron microscopy (SEM) was used to monitor the fabrication process and investigate the morphology and dimensions of the structures, using a Hitachi S-4300 SEM.
